# Targeting the ATR-CHK1 Axis in Cancer Therapy

**DOI:** 10.3390/cancers9050041

**Published:** 2017-04-27

**Authors:** Stuart Rundle, Alice Bradbury, Yvette Drew, Nicola J. Curtin

**Affiliations:** 1 Northern Institute for Cancer Research, Newcastle University, Newcastle upon Tyne NE2 4HH, UK; a.bradbury@newcastle.ac.uk (A.B.); yvette.drew@newcastle.ac.uk (Y.D.); 2 Northern Gynaecological Oncology Centre, Queen Elizabeth Hospital, Gateshead NE9 6SX, UK; 3 Northern Centre for Cancer Care, Freeman Hospital, Newcastle upon Tyne NE7 7DN, UK

**Keywords:** ATR, CHK1, cell cycle, chemotherapy-sensitising-agents, DNA-damage, DNA-repair, protein-kinase-inhibitors, radiation-sensitising-agents

## Abstract

Targeting the DNA damage response (DDR) is a new therapeutic approach in cancer that shows great promise for tumour selectivity. Key components of the DDR are the ataxia telangiectasia mutated and Rad3 related (ATR) and checkpoint kinase 1 (CHK1) kinases. This review article describes the role of ATR and its major downstream target, CHK1, in the DDR and why cancer cells are particularly reliant on the ATR-CHK1 pathway, providing the rationale for targeting these kinases, and validation of this hypothesis by genetic manipulation. The recent development of specific inhibitors and preclinical data using these inhibitors not only as chemosensitisers and radiosensitisers but also as single agents to exploit specific pathologies of tumour cells is described. These potent and specific inhibitors have now entered clinical trial and early results are presented.

## 1. Introduction

Dysregulation of the DNA damage response (DDR) provides the genomic instability that is an enabling characteristic of cancer [1], however, this also provides opportunities for exploitation with inhibitors of complementary DDR pathways on which the cancers have become dependent [2]. This principle has been clearly demonstrated pre-clinically and clinically with PARP inhibitors that target base excision DNA repair in cancers defective on homologous recombination DNA repair, culminating in the approval of three PARP inhibitors for ovarian cancer therapy [3].

Probably the most common defect in cancer is loss of G1 cell cycle checkpoint control, often, but not exclusively, due to defects in the p53-Rb pathway [4]. Their resulting reliance on the S and G2 checkpoints may be exploited by targeting the ataxia telangiectasia mutated and Rad3 related (ATR) and checkpoint kinase 1 (CHK1) kinases, which are the key kinases linking DNA lesions to cell cycle checkpoints and repair. This review describes the role of ATR and CHK1 and their validation as targets for cancer therapy, the development of inhibitors as chemo and radiosensitisers and as single agents exploiting the molecular pathology of cancer through to early clinical trials.

## 2. The Role of ATR and CHK1 in the DDR

### 2.1. ATR and CHK1 Activation by DNA Damage

**Figure 1:**
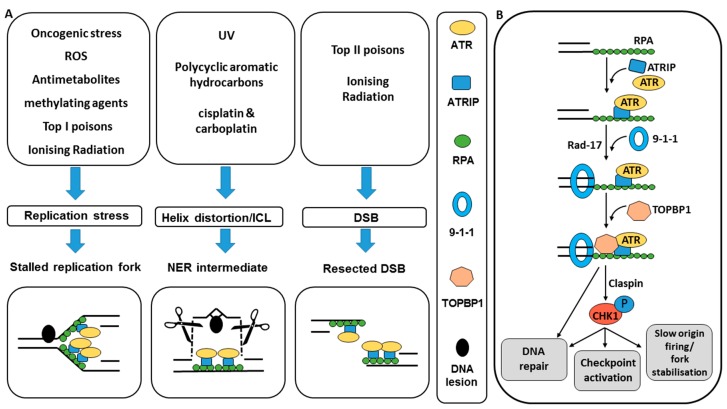
Induction of ATR-CHK1 pathway activation and downstream signalling. (**A**) Anti-metabolites such as hydroxyurea (HU) and gemcitabine deplete the dNTP pool and cause stalled replication forks [13,14,15]. Topoisomerase 1 poisons and monofunctional DNA alkylating agents cause single strand lesions that can also stall/collapse replication forks. This increases replication stress and the availability of ss-DNA for ATR activation. Poly-cyclic aromatic hydrocarbon induced bulky DNA adducts and intra-strand cross-links resulting from agents such as UV and platinum based chemotherapy drugs are repaired by NER, leaving behind a short strand of ss-DNA [16,17]. The ssDNA is also present at the site of IR or topoisomerase II poison induced DSBs that have been resected by exo- or endo-nucleases [18]. In all cases the ssDNA is first coated by RPA. (**B**) RPA enables localisation of ATR to sites of DNA damage [10]. ATR recognition of the RPA-ssDNA complex is dependent on ATR-interacting protein (ATRIP). Localisation of the 9-1-1 complex via RPA interaction with RAD17 and subsequent recruitment of TOPBP1 and claspin leads to ATR activation and subsequent phosphorylation events leading to cell cycle arrest and DNA repair [9,15]. CHK1 acts as an intermediary in many of the DNA repair and DNA checkpoint reactions resulting from the activation of ATR and contributes to fork stabilisation and inhibiton of replication origin firing.

The DNA damage response (DDR) is critical to protect cells from the high levels of DNA damage sustained on a continuous basis. The vast majority of this damage is unavoidable as it is generated endogenously (e.g., reactive oxygen species) or a result of normal environmental exposure (e.g., UV). The DDR is a highly coordinated system involving cell cycle checkpoints, which prevent damage becoming fixed by DNA replication or passed on to daughter cells at mitosis and provide the necessary pause for repair, and the DNA repair pathways themselves. Ataxia telangiectasia mutated and Rad3 related (ATR) kinase is a PIKK family member with similar structure to ataxia telangiectasia mutated (ATM) and DNA-PKcs that also have key roles in the DDR. The principal phosphorylation target of ATR is checkpoint kinase 1 (CHK1) and the function of both of these kinases is critical to cell cycle control and the maintenance of genomic integrity in response to DNA damage and replication stress. Homozygous disruption of ATR or CHK1 is lethal in early embryonic life [5,6], underlying the critical role that these protein kinases play. The development of kinase dead ATR (ATR-KD) cells, where an inactive form of ATR acts as a dominant negative inhibitor to native ATR function led to the demonstration that ATR-KD cells were sensitive to DNA damaging agents and did not arrest at the G2/M checkpoint (see Section 3), suggesting a role for ATR in both DNA damage repair and cell cycle checkpoint regulation [7]. The principal activator of the ATR-CHK1 pathway is replication stress that is common in cancers, particularly those with activated oncogenes and dysfunctional G1/S checkpoint control [8]. At the molecular level ATR is activated by the presence of single stranded DNA (ssDNA) that arises out of stalled replication forks, nucleoside excision repair (NER) intermediates or resected DSBs that have been subject to exonuclease digestion [9,10,11]. There is some evidence that the level of ATR activation is dependent of the amount of replication protein A (RPA) and therefore the length of ssDNA that is present [12]. Lesions that result in the presence of a length of ssDNA result from a wide variety of exogenous and endogenous DNA damaging agents (Figure 1A).

In all eukaryotic cells, ssDNA is first sensed by RPA. RPA enables localisation of ATR to sites of DNA damage [10]. ATR recognition of the RPA-ssDNA complex is dependent on ATR-interacting protein (ATRIP). ATRIP is crucial to the functioning of ATR such that there are no phenotypical differences in organisms that have lost ATR or this obligate subunit [13,14]. Activation of the ATR-CHK1 pathway requires the additional localisation of RAD9, RAD1, HUS1 (which forms the hetero-trimeric ring shaped complex known as 9-1-1) via RPA interaction with RAD17. The 9-1-1 complex recruits topoisomerase binding protein-1 (TOPBP1) and it is thought that TOPBP1 binding is the critical step in ATR activation and subsequent phosphorylation events [9,15]. The primary phosphorylation target for ATR kinase is checkpoint kinase 1 (CHK1), which acts as an intermediary in many of the DNA repair and DNA checkpoint reactions that result from the activation of ATR at sites of DNA damage (Figure 1B).

### 2.2. ATR and CHK1 Signal to DNA Damage Checkpoints

Once activated and localised at the site of the DNA lesion, ATR signals to coordinate cell checkpoint control and DNA repair, which is undertaken by a large number of ATR substrates. Crucial for regulating both cell cycle control at the G2/M checkpoint and DNA replication is the ATR-CHK1 pathway. CHK1 is transiently located at the site of DNA damage and is activated by phosphorylation at two sites by ATR, Ser317 and Ser345 [16,17,18]. CHK1 phosphorylation by ATR requires Claspin to act as a “mediator” protein, bringing ATR and CHK1 together [19,20]. Claspin is localised to the DNA lesion by RAD17 (also required for recruitment and loading of the 9-1-1 complex at the site of DNA damage) and is activated when bound to its phosphorylated form. This phosphorylation of RAD17 is ATR dependent [21]. Activated, phosphorylated CHK1 is released from chromatin and signals DNA damage to the rest of the nucleus. Uncertainty remains over whether it is this sub-cellular re-distribution of activated CHK1 or increased catalytic activity that results in checkpoint reactions [22].

Important CHK1 targets are Wee1 and the cell division cycle proteins (cdc) cdc25A and cdc25C. Phosphorylation of cdc25 proteins and Wee1 results in inhibition of cyclin-dependent kinase (CDK1/CDK2) activity. Phosphorylation of cdc25A results in inhibition of CDK2 resulting in S-phase arrest, and phosphorylation of cdc25C and Wee1 causes inhibition of CDK1, resulting in G2/M arrest [23,24,25,26] and it is through this function that the ATR-CHK1 pathway exerts cell cycle control at the G2/M and intra-S cell cycle checkpoints [9], preventing entry to mitosis via inhibition of CDK1 thereby preventing immediate mitotic catastrophe or permanent loss of genetic material. 

### 2.3. ATR and CHK1 Signal to Reduce Replication Stress

ATR-CHK1 signaling inhibits replication origin firing, thus reducing the rate of DNA replication under replication stress and DNA damaging conditions. The precise biochemical pathways whereby ATR exerts this effect are not fully understood but appear intimately related to the reactions which initiate and maintain the intra-S checkpoint [9,27]. CHK-1 mediated Wee-1 activation and cdc25A inhibition result in inhibition of CDK2 catalytic activity and a slowing in the rate of DNA synthesis through late replication origin suppression [28]. When replication is halted due to a lesion blocking fork progression or severely limited supply of dNTPs replication fork progression is prevented and the paused replication forks may stall. Stabilisation of stalled replication forks is dependent on the presence of functional ATR signalling through CHK1 and their absence results in replication fork ‘collapse’ [29], however exactly how CHK1 prevents or slows replication fork collapse during periods of replication stress is unclear.

### 2.4. ATR and CHK1 Signal to DNA Repair

In addition to the well-established regulation of cell cycle checkpoints and signalling to DNA replication via CHK1 kinase, a further class of ATR substrates includes those that function to regulate DNA repair. ATR targets the Fanconi-anaemia proteins, FANCD2 and FANCI to regulate inter-strand crosslink repair [30]: ATR phosphorylation of FANCD2 promotes its localisation to DNA damage foci through monoubiquitination [31]. ATR also phosphorylates the NER protein XPA, regulating its recruitment to DNA lesions [32].

ATR and CHK1 appear to be intimately involved in the regulation of DNA repair by homologous recombination DNA repair (HRR). Early studies showed that ATR phosphorylated and activated the key HRR regulatory protein BRCA1 [33]. CHK1 also recruits and phosphorylates two key HRR proteins: RAD51 recombinase and BRCA2 [34]. Cells treated with caffeine and NU6027, early inhibitors of ATR, or inhibitors of CHK1 display reduced RAD51 repair foci in response to treatment with DNA damaging hydroxyurea (HU) [34,35,36,37]. RAD51 foci are a long-accepted marker for HRR function [38]. Very recently the role of ATR in coupling HRR with cell cycle checkpoints has been further elucidated by demonstrating that ATR enhances BRCA1-PALB2 binding to promote HRR is at least in part due to inhibition of CDK activity [39]. ATR and CHK1 therefore play an important role in the maintenance of DNA integrity in the face of DNA damaging insults principally through their involvement in HRR as well as cell cycle checkpoints.

## 3. Validation of Target and Rationale for Cancer Specificity

Genomic instability is recognised as an enabling characteristic of cancer [40] and a major contributor to this characteristic is considered to be impairment of cell cycle checkpoints [41]. Cancer cells are often defective in their G1 checkpoint control due to mutations in the tumour suppressor genes p53 and pRb, or an imbalance in cyclins, cyclin-dependent kinases (CDKs) and their inhibitors [4,42]. Coupled with frequent activation of oncogenes that drive replication (e.g., Myc, RAS, etc.) [43] cancer cells are much more likely to enter S phase with increased replicative stress, stalled replication forks and generate replication-associated DSBs [44]. They therefore tend to be more reliant than normal cells on their S and G2 checkpoints to ensure this damage is not transmitted further. As a result of this the S/G2 checkpoint and thus the ATR/CHK1 pathway is an attractive target for cancer-specific therapy.

It is well known that ATR and CHK1 are essential protein kinases; homozygous deletion of ATR or CHK1 leads to peri-implantation embryonic lethality [5,6], and no living human has been identified as completely lacking either ATR or CHK1 function. However Seckel syndrome, which is characterised as having low levels of ATR expression due to hypomorphic mutation of the ATR gene results in growth retardation and microcephaly [45]. Although ATR^+/−^ mice have increased incidence of tumour formation, Seckel syndrome sufferers do not have an increased prevalence of cancer [6].

Early validation studies of ATR and CHK1 as anticancer targets were done by genetic means. For ATR inactivation studies, induced expression of a dominant negative ATR-KD (kinase dead) mutant rendered cells sensitive to a wide variety of DNA-damaging agents, including IR, alkylating agents (methyl-methane sulfonate), DNA cross-linking agents (cisplatin), topoisomerase I and II poisons (topotecan, SN-38, etoposide, doxorubicin) and antimetabolites (hydroxyurea) [7,46,47]. CHK1 inactivation studies were mainly carried out by siRNA or shRNA knockdown of CHK1 expression, which also sensitised cells to a variety of DNA damaging agents: topoisomerase I and II poisons (SN-38, etoposide, doxorubicin), antimetabolites (cytarabine, 5-fluorouracil, gemcitabine) and enediyne anticancer antibiotics (lidamycin) [48,49,50,51,52,53]. However, unlike ATR downregulation, CHK1 downregulation did not sensitise HCT-116, HeLa or U2OS cells to cisplatin [53].

In some studies, the downregulation of either ATR or CHK1 sensitised p53-deficient cells significantly more than p53-proficient cells leading to the idea that targeting the pathway would be particularly effective in cells with defective p53. For example, downregulation of CHK1 and ATR sensitised p53-deficient HCT-116 cells to DNA damage to a greater extent than HCT-116 p53-wild type cells [50,51,54]. However, in marked contrast, impairment of CHK1 by UCN-01 or siRNA caused similar sensitisation of paired U2OS cells with wt p53 or p53 knockdown to irinotecan and cisplatin, [54]. Similarly in the study by Flatten et al. [49] ATR downregulation by siRNA sensitised both U2OS (wildtype p53) and HeLa (p53 defective) cells to the topoisomerase I poisons camptothecin and SN-38. Furthermore, in U2OS cells with impaired G1 checkpoint control for various reasons relevant to cancer (e.g., overexpression of cyclin D1, cyclin E, CDK2, MDM2 or human papilloma virus E2), expression of ATR-KD or treatment with caffeine augmented the sensitisation of cells to DNA damage. In these cells, reinforcing G1 control through induced expression of p21 or p27 prevented sensitization [47]. Thus, it is likely that loss of G1 control in general, rather than just p53, leads to greater sensitisation following impairment of ATR/CHK1 signalling.

## 4. Development of ATR and CHK1 Inhibitors

Depletion of ATR or CHK1, e.g., using siRNA as described above, enhanced tumour cell killing by a wide range of genotoxic agents. Similar results have been obtained with prototype small molecule inhibitors of ATR and CHK1 some of which, such as UCN-01, were originally developed with other molecular targets in mind [55]. Early inhibitors were non-specific but more recently potent and selective inhibitors of both ATR and CHK1 have been developed. A number of these compounds, with a focus of those for which pre-clinical data is available and described later in this review are outlined, below (see Table 1).

### 4.1. CHK1 Inhibitors

The dual CHK1/CHK2 inhibitor AZD7762 has been used extensively in pre-clinical studies (see Section 5) and was developed using structure based drug design, following the identification of thiophene carboxamide ureas as inhibitors of CHK1 by high throughput screening [56]. V158411 is another example of a small molecule inhibitor of CHK1 that had similar inhibitory activity against CHK2 in biochemical assays but was 20× more selective for CHK1 in cell-based studies. V158411 was developed by elaboration of a fragment core using structure based design, this drug was the culmination of efforts to develop a series of potent CHK1 inhibitors [57]. 

PF477736 was amongst the first of the truly selective CHK1 inhibitors described, with an approximate 100-fold selectivity ratio for CHK1 versus CHK2 [58]. Although extensive pre-clinical characterisation and activity data is available, details of the drugs development are limited. Following identification of a candidate pyrazole[1-5-a]pyrimidine compound with CHK2 inhibitory activity then a combination of a cell-based functional screening assay for γ-H2AX induction, and medicinal chemistry exploration techniques MK8776 (formerly known as SCH900776) was developed as a highly potent and selective CHK1 inhibitor with a 500-fold selectivity for CHK1 versus CHK2 [59,60].

Pyrazine compounds were identified as potential inhibitors of CHK1 by virtual and high throughput biochemical screening for fragment hits and structure based drug design that led to the development of the CHK1 specific inhibitor CCT244747 with a greater than 1000-fold selectivity for CHK1 over CHK2 [60,61]. CCT244747 was also the first selective CHK1 inhibitor available with oral bioavailability and was followed by the related orally bioavailable clinical development candidate CCT245737 (CHK1 IC_50_ = 1.3 nM, CHK2 IC_50_ = 2440 nM) described by the same development group [62]. Another pyrazine compound LY2603618 has shown high specificity for CHK1 vs. CHK2 and hasoral bioavailability (CHK1 IC_50_ = 7 nM, CHK2 IC_50_ = 12,000 nM) [63]. The related second generation inhibitor, LY2606368 has entered clinical trials [64]. As with many commercially developed drugs, data specifying the details of its design and development are limited.

### 4.2. ATR Inhibitors

The development of inhibitors with potent and specific inhibition of ATR has lagged behind that of CHK1 inhibitors, possibily because of the difficulty in obtaining pure active protein for an in vitro activity assay. The natural product, caffeine, was observed to sensitise cells to UV induced DNA damage and abrogate the G2/M checkpoint, but it was weak (ATR IC_50_ = 1.1 mM) and inhibition of other DDR proteins such as ATM, DNA-PKcs and mTOR was also observed [65]. Another natural product, Schisandrin B was identified as a more potent inhibitor of ATR (ATR IC_50_ = 7.3 µM) through a cell-based screen of herbal compounds. Importantly, inhibition of other DDR proteins (ATM, DNA-PKcs, mTOR, PI-3K) was not observed with this compound [66] making its observed abrogation of UV induced G2/M checkpoint induction more reliably attributable to ATR inhibition. Cell based screening for potent and specific ATR inhibitors has been limited by the fact that its kinase activity is limited to the S and G2 phase of the cell cycle, meaning that a large number of false positives were likely to be identified from an indirect effect of the tested compound on the cell cycle [67]. NU6027 was developed as a CDK2 inhibitor but its potentiation of cisplatin toxicity in breast and ovarian cancer cell lines led to its investigation as an inhibitor of ATR. NU6027 was found to be a more potent inhibitor of ATR than of CDK2 (ATR IC_50_ = 1 nM) [35].

The development of a novel screening platform in which pan-nuclear induction of phosphorylation of H2AX was strictly dependent on ATR, and independent of ATM or DNA-PKcs, provided a reliable indicator of ATR activity to test candidate compounds. Using this screening platform to test a pool of PI-3K inhibitors (which, due to the similarity of PI-3K and other PIKKs, were likely to be enriched for potential ATR inhibitors) the first highly potent and specific ATR inhibitor, ETP-46464 (ATR IC_50_ = 25 nM) was identified [67]. Poor pharmacokinetic properties, however prevented the further development of ETP-46464 into a viable clinical candidate. Further high throughput screening using ATR-specific kinase assays has enabled the development of potent and specific ATR inhibitors with the potential for clinical development. A high throughput screening hit and subsequent structural modification of an aminopyrazine precursor led to the development of VE-821. Structural modification of the precursor compound led to increased potency against ATR (ATR IC_50_ = 26 nM), a concomitant increase in ATM and DNA-PK inhibitory activity was also observed. Despite this, the selectivity of VE-821 for ATR remains over 100-fold versus that of its other targets [68]. Extensive pre-clinical in vitro data exists for this compound as detailed in Section 5 of this review. The VE-821 analogue, VE-822 (now in clinical trials as VX-970) has increased potency (ATR IC_50_ = 0.2 nM) and maintains >100-fold selectivity for ATR over ATM and other protein kinase targets [69]. Favourable in vivo and toxicological properties have led to the development of VX-970 as the first selective ATR inhibitor to be entered into clinical trials (see Section 8 below). The specifics of the structural development of VX-970 have not yet been published though it was developed from the same aminopyrazine screening hit as its analogue, VE-821 [70]. High throughput screening of another series of compounds with structural similarity to known PIKK inhibitors identified a precursor morpholine class compound as an inhibitor of ATR. Structural modification to enhance potency and specificity for ATR led to the development of AZ20 (ATR IC_50_ = 5 nM) [71]. The main limitations of AZ20 that have prevented its progression to clinical studies include poor aqueous solubility, a property that has been overcome by the related sulfoximine morpholinopyrimidine AZD6738. AZD6738 is a specific and orally bioavailable ATR inhibitor that has progressed to clinical trials (ATR IC_50_ = 1 nM) [70], though again the specifics of its development are not published.

## 5. Pre-Clinical Data: Chemo- and Radio-Sensitisation In Vitro and In Vivo

Many standard chemotherapy agents cause replication stress, antimetabolites and topoisomerase I poisons in particular, or, like radiotherapy, cause DNA lesions that activate ATR/CHK1 signalling. ATR and CHK1 inhibitors have therefore been evaluated as sensitisers of ionising radiation (IR) and several cytotoxic drugs in a variety of models of human cancer. Biological evaluation of the ATR and CHK1 inhibitors in vitro and in vivo has revealed interesting similarities and differences in their activity in combination with chemo or radiotherapy or molecularly targeted drugs and as single agents in cells with distinct molecular pathologies.

Studies using early inhibitors such as UCN-01 and NU6027 provided much proof of principle and mechanistic evidence for the use of small molecule inhibitors of CHK1 and ATR, respectively in an anti-cancer setting [35,72]. Due to a lack of target specificity it was difficult to attribute the effects of these drugs to inhibition of ATR or CHK1 and this led to efforts to develop more potent and selective inhibitors that are the focus of this section of the review.

### 5.1. Combinations with Chemotherapy Agents

#### 5.1.1. ATR and CHK1 Inhibitors in Combination with Antimetabolite Drugs In Vitro

The CHK1/2 inhibitor, AZD7762 was shown to sensitise human cancer cells to gemcitabine and topoisomerase poisons [73] and it is with gemcitabine that the most consistent and potent chemo-potentiation has been observed in vitro amongst this class of inhibitors. AZD7762 potentiated the cytotoxicity of gemcitabine in a panel of NSCLC cell lines [74] and also in a panel of neuroblastoma cells lines [75]. In both cases sensitisation to gemcitabine was observed to be independent of their p53 status or G1 checkpoint proficiency and this observation is consistent with the demonstration that chemosensitisation by CHK1 knock down was not specific to p53 deficient cells [54]. Chemosensitisation by the more selective CHK1 inhibitors may be more dependent on the p53 status of the tumour cells but many studies were conducted in only p53 dysfunctional cells, without p53 functional ones as control, so the dependence on p53 dysfunction cannot be conclusively ascertained. For example, MK8776 potentiated gemcitabine cytotoxicity in a variety of p53 mutant cell lines including those derived from TNBC [76]. Similarly, CCT244747 abolished gemcitabine-induced G2/M arrest and potentiated its cytotoxic effects in p53 mutant colon, lung and pancreatic cancer cell lines [61] but was not evaluated in p53 wt cells. However, MK8776 potentiated gemcitabine cytotoxicity only in pancreatic cancer cells that were p53 deficient [77]. Likewise, V158411 potentiated gemcitabine cytotoxicity in p53 mutant, but not wild type, TNBC and ovarian cancer cell lines [78] and in a panel of p53 mutant colon, lung and prostate cancer cell lines, whereas unmatched p53 proficient cell lines were unaffected [57]. In a report using LY2603918, p53 mutant colon cancer cells were sensitised to gemcitabine but LY2603918 did not affect survival of their matched p53 wild type parental cell line [63].

Other anti-metabolite drugs have been used in combination with CHK1 inhibitors for in vitro studies. Incorporation of cytarabine into DNA activates CHK1, which stabilizes stalled replication forks, induces S-phase slowing, and diminishes cytarabine cytotoxicity. MK8776 caused a 20–70 fold potentiation of both hydroxyurea and cytarabine in triple negative breast cancer (TNBC) cell lines [76]. MK8776 also potentiated cytarabine in human AML cell lines and patient AML samples ex vivo [79]. Modest potentiation of cytarabine cytotoxicity in complex karyotype AML blast cell cultures was also seen in co-treatment with AZD7762 [80].

Similarly, ATR inhibitors have also shown significant synergy in combination with anti-metabolite agents, particularly gemcitabine, although fewer studies have been conducted. The first truly selective ATR inhibitor, VE-821, showed synergy with gemcitabine in human colon cancer cell lines [81]. Its analogue, VE-822, now in clinical development as VX-970, also sensitised lung cancer cell lines to gemcitabine toxicity. This effect was greater than that seen in combination with topoisomerase inhibitors or oxaliplatin and equalled only by cisplatin [82].

#### 5.1.2. ATR and CHK1 Inhibitors in Combination with Topoisomerase Poisons In Vitro

Both ATR and CHK1 inhibitors have shown synergy with topoisomerase poisons in vitro and in vivo. The CHK1/2 inhibitor, AZD7762, potentiated topotecan cytotoxicity in G1 checkpoint defective neuroblastoma cell lines, but in contrast to the results seen in combination with gemcitabine, cell lines with an intact G1 checkpoint were not affected [75]. A 10-fold potentiation of topotecan was observed in high grade serous ovarian cancer cell lines when used in combination with PF477736 [83]. In this report, all cell lines were p53 deficient, as is common in ovarian cancer. The ATR inhibitor VE-821 enhanced the cytotoxicity of camptothecin and an experimental topoisomerase I poison, LMP400, in colon and breast cancer cells, with a greater effect seen in p53 mutant variants [84]. The clinically active ATR inhibitor, VE-822, also potentiated etoposide and the topoisomerase I poison, SN-38, in a panel of lung cancer cells, though not to the extent seen with gemcitabine and cisplatin [82].

#### 5.1.3. ATR Inhibitors in Combination with Platinum-Based Chemotherapy Agents In Vitro

The DNA cross-linking platinum complexes, cisplatin and carboplatin are widely used anti-cancer drugs. In particular, they are important in the treatment of a number of solid organ cancers including lung, testicular and colon cancer and form the mainstay of systemic drug treatment of cervical and ovarian cancer. Intra-strand crosslinks that result from treatment with these agents are repaired by NER and impede the progress of the replication fork, both of which activate ATR [85,86,87]. Platinum complexes are particularly cytotoxic to cells lacking HRR [88], which is dependent on the ATR/CHK1 pathway, furthermore, cisplatin-induced DNA damage activates ATR kinase and subsequent downstream targets [89].

ATR inhibitors demonstrate substantial potentiation of cisplatin. In fact one of the earliest observations made with the prototype ATR inhibitor, caffeine, was cisplatin potentiation, which was found to be dependent on replication [90]. Subsequent experiments established that cisplatin treatment of these cells resulted in protracted G2 arrest followed by aberrant mitosis and apoptosis. Caffeine abrogated the G2 arrest and accelerated the progression to mitosis, resulting in increased cell death [87]. Caffeine was subsequently shown to be acting via ATR inhibition and perturbation of the G2/M checkpoint [91]. Similarly, the early ATR inhibitor, NU6027 was investigated in combination with several cytotoxic drugs and the greatest synergy was observed with cisplatin [35] In this report greater sensitisation of cisplatin was observed in cells with wild type p53, although this was based on one pair of cell lines and the p53 mutant one was also cisplatin-resistant by virtue of a defect in mismatch repair.

The potent and selective ATR inhibitor, VE-821, caused up to 10-fold potentiation of cisplatin and carboplatin in p53 mutant or ATM deficient human colon cancer cells [81]. Consistent with this was the observations that knockdown of p53 by siRNA or expression of human papillomavirus E6 protein (which targets p53 for degradation) was shown to enhance the synergy between VE-821 and cisplatin [65]. The combination of VE-821 and cisplatin was also shown to synergise in ATM-null, but not normal skin fibroblast [81]. VE-822 (VX-970) sensitised lung cancer cell lines to cisplatin to a similar degree as that seen in combination with gemcitabine, with the greatest effect again seen in p53 deficient cell lines [82]. Similarly, AZD6738 caused a concentration-dependent synergy with cisplatin in *K-ras* mutant, ATM deficient NSCLC cell lines [92] and ETP-46464, another ATR inhibitor substantially increased cisplatin cytotoxicity in a panel of ovarian, endometrial and cervical cancer cell lines. In this study ATM inhibition did not further enhance cisplatin potentiation by ETP-46464 [93].

#### 5.1.4. CHK1 Inhibitors in Combination with Platinum Agents In Vitro

It has been suggested that ATR but not CHK1 activity is required for resistance to cisplatin [94] and inconsistency in the effects of CHK1 inhibition as a strategy for enhancing the cytotoxicity of platinum drugs has been observed. While AZD7762 reversed cisplatin resistance in NSCLC cell lines, independently of their p53 status [74] potentiation of cisplatin cytotoxicity in neuroblastoma cell lines was only seen in those that were G1 checkpoint defective (by p53 mutation, MDM2 amplification or p14 deletion) [75]. Cisplatin resistance has also been overcome by AZD7762 in a panel of clear cell ovarian cancer cell lines [95] and in p53 mutant HNSCC cells [96]. However, MK 8776 failed to sensitise p53 mutant TNBC cells to cisplatin treatment [76] and, although V158411 did potentiate the cytotoxic effects of cisplatin and carboplatin in a number of TNBC and ovarian cancer cell lines in a p53 dependent manner [78], the effect was significantly less than in combination with gemcitabine. Similarly, V158411 potentiation of cisplatin in p53 deficient lung, colon and prostate cancer cell lines was also less that that seen with gemcitabine [57].

#### 5.1.5. CHK1 Inhibitors in Combination with Taxanes In Vitro

Curiously, CHK1 inhibitors have been reported to enhance the cytotoxicity of the taxanes, which are antitubulin agents rather than DNA damaging agents. The CHK1 inhibitor, PF477736, enhanced docetaxel cytotoxicity in colon cancer cell lines [97]. The mechanism was proposed to be via modulation of docetaxel-induced changes in phosphorylated histone H3 and Cdc25C, suppressing M-phase arrest and sensitising the cells to docetaxel-induced apoptosis. Similarly CCT244747 suppressed paclitaxel-induced histone H3 phosphorylation in HNSCC cell lines though the combination of paclitaxel and the CHK1 inhibitor was not synergistic in cell killing [98].

#### 5.1.6. ATR and CHK1 Inhibitor- Cytotoxic Drug Combinations In Vivo

In-vivo studies combining ATR or CHK1 inhibitors with chemotherapy agents have largely confirmed the increased anti-tumour activity predicted by the in vitro data, outlined above. The ATR inhibitor VE-822 (VX-970), although it had no single-agent activity on the schedule used, significantly enhanced the efficacy of cisplatin in six out of seven mice xenograft models of lung tumours without an increase in toxicity, as measured by weight loss, over cisplatin treatment alone [82]. Remarkably, the combination led to complete tumour growth inhibition in the three cisplatin insensitive models and complete tumour regression in one cisplatin sensitive model that persisted for three weeks following cessation of treatment. Potentiation of cisplatin-induced tumour growth delay by AZD6738 was observed in mice bearing xenografts of human NSCLC tumours [92]. Whilst neither AZD6738 (daily × 14) nor cisplatin (days 1 and 8) alone caused significant tumour growth delay, the combination inhibited tumour growth by 75.5% and this effect was greater in ATM deficient tumours (84.8%). Again, no significant increase in toxicity was observed with combination treatment over cisplatin, alone.

VE-822 (VX-970) potentiated the antitumour activity of the topoisomerase I inhibitor, irinotecan, in mice bearing human colon cancer xenografts [84]. Mice were treated with IP irinotecan on day 0 of a 4 days cycle and oral VX-970 on three consecutive days. The combination with VX-970 significantly increased the antitumour activity of irinotecan without substantially increasing irinotecan toxicity. The ATR inhibitor was reported to have been tolerable with no additional toxic effects observed over irinotecan alone.

CHK1 inhibitors have also been studied in a variety of tumour models and drug combinations. AZD7762 potentiated the anti-tumour activity of gemcitabine in G1/S checkpoint defective neuroblastoma xenografts [75] with the antitumor activity of the combination being significantly greater than either AZD7762 or gemcitabine alone and without a significant difference in the tolerability of the regimes as determined by weight loss. In mice bearing NSCLC xenografts, co-treatment of AZD7762 with gemcitabine or cisplatin significantly reduced tumour growth rate compared to either gemcitabine or cisplatin alone with protracted tumour growth inhibition being observed for three weeks following cessation of treatment [74]. Synergistic activity was also demonstrated with AZD7762 in combination with cisplatin in xenograft models of clear cell ovarian cancer [95]. As with the data for combinations with ATR inhibitors, CHK1 inhibitors in combination with cytotoxic drugs have not been observed to significantly increase the toxicity of the cytotoxic drug treatment.

The CHK1 inhibitor, V158411, also enhanced irinotecan-induced tumour growth delay in colorectal tumour xenografts [57]. Tumour growth delay with the combination was 203% compared with only 79% with irinotecan alone and the combination was no more toxic than irinotecan alone. Similarly, co-administration of CCT245737 doubled the tumour growth delay observed with irinotecan alone in human colorectal mouse xenografts [62].

The surprising observation that CHK1 inhibitors potentiated taxane activity in vitro has also been observed in vivo. PF477736 significantly extended docetaxel-induced tumour growth delay in mice bearing colon cancer xenografts and caused complete remission in 3 of 12 mice, whereas all tumours eventually relapsed in mice treated with docetaxel alone. PF477736 did however cause a dose dependent increase in weight loss that recovered during the course of the experiment [97]. Oral CCT244747 administered on alternate days for 3 doses with concurrent IP paclitaxel to mice bearing HNSCC xenografts, however failed to enhance tumour growth inhibition without the addition of IR as a triple therapy [98].

### 5.2. Combinations with Ionising Radiation (IR)

#### 5.2.1. Radiopotentiation and Chemo-Radiopotentiation by CHK1 Inhibitors In Vitro

Ionising radioation (IR) causes a plethora of DNA damage, and is a potent inducer of the ATR-CHK1 response. Radiosensitisation by CHK1 inhibitors has been investigated in a number of models. In pancreatic cancer cells low dose AZD7762 abrogated the G2 checkpoint, inhibited RAD51 focus formation, increased the persistence of γ-H2AX expression and reduced cell survival with a radiation enhancement ratio of 1.5 at the LD_90_ [37]. Co treatment of lung cancer cell lines with IR and AZD7762 resulted in increased ATR/ATM mediated CHK1 phosphorylation, stabilised cdc25A and suppressed cyclin A expression, reducing survival in clonogenic assays [99]. Non-cytotoxic concentrations of AZD7762 also enhanced the radio-sensitivity of a panel of colon, prostate, lung, pancreas and glioblastoma cell lines in clonogenic survival assays and this effect was greater in p53 deficient cells [100]. In contrast, a wide panel of human cancer cell lines was sensitised to IR by AZD7762 independently of their p53 and BRCA2 status [101]. However, AZD7762 and LY2603618 did not increase the radiosensitivity of radioresistant breast cancer cells, with high levels of oncogene and DDR proteins, or their radiosensitive parental cell lines [102]. Both drugs suppressed cell growth in the radio-resistant but not the radiosensitive cell lines when used as a single agents, supporting evidence for a role for CHK1 inhibitors in cells with high levels of endogenous replicative stress (see Section 7).

Combining CHK1 inhibitors with IR and cytotoxic drug dual treatment may further enhance cytotoxicity. AZD7762 radiosensitised a panel of p53 mutant colon cancer cell lines to a wide range of IR doses. Sensitisation was further enhanced by the addition of 5-FU, significantly reducing the surviving fraction of cells [103]. When combined with gemcitabine, AZD7762 significantly enhanced the cytotoxic effects of IR on pancreatic cancer cells when compared to a combination of either drug used singly with IR [37].

#### 5.2.2. Radiopotentiation and Chemo-Radiopotentiation by CHK1 Inhibitors In Vivo

In the in vivo setting, AZD7762 successfully sensitised pancreatic cancer cell line and patient derived xenograft models to gemcitabine-radiation combination therapy, retarding tumour growth relative to gemcitabine-radiation combination therapy by >35 days. Although there was a trend for AZD7762 to sensitize tumours to radiation alone it was not statistically significant [37]. AZD7762 increased the tumour growth delay following fractionated radiation from 7.4 to 18.7 days in human colorectal adenocarcinoma xenograft models [100]. In xenograft models of lung cancer brain metastasis, AZD7762 significantly prolonged the median survival time in response to radiation [99]. In mice bearing HNSCC xenografts the antitumour activity of IR plus paclitaxel was significantly potentiated by CCT244747 without further increasing toxicity [98].

#### 5.2.3. Radiopotentiation and Chemo-Radiopotentiation by ATR Inhibitors In Vitro and In Vivo

ATR inhibitors have also been shown to increase the cytotoxicity of IR alone or in combination with other cytotoxic drugs. VE-821 caused significant enhancement of IR-induced cell death in a wide variety of human cancer cell lines, which included cervical, colon, TNBC and HNSCC cancer [104]. This report also showed that radio-sensitisation using VE-821 was observed under hypoxic conditions, a finding that is significant as hypoxic cells tend to show an aggressive phenotype that is often resistant to both chemotherapy and radiotherapy [105,106]. It should be noted however that this effect is yet to be replicated in vivo. VE-822 (VX-970) potentiated the effects of both gemcitabine and IR in pancreatic ductal adenocarcinoma cells with the triple combination of gemcitabine, radiation and VE-822, causing the greatest reduction in clonogenic survival. This effect was also demonstrated in xenograft models. VE-822 enhanced the efficacy of single dose and fractionated IR, sensitising pancreatic ductal carcinoma cell xenografts to IR alone and in combination with gemcitabine. Addition of VE-822 at a non-toxic dose resulted in a significant delay in tumour growth rate compared to IR alone over a range of dosing schedules and this effect was further enhanced with the addition of gemcitabine, again with no weight loss or other increased toxicity reported for the triple combination [69].

## 6. Combinations with Other Molecular Targeted Agents

The potential of combining ATR and CHK1 inhibitors with other agents targeted at the DDR has gained considerable attention. Three examples have been described, ATR or CHK1 inhibition with PARP inhibition, ATR inhibition with CHK1 inhibition and CHK1 inhibition with Wee1 inhibition.

PARP inhibition is synthetically lethal in combination with loss of the HRR-genes BRCA1 and BRCA 2. Therefore it was predicted that loss of ATR and/or CHK1 would lead to a BRCA-like phenotype, sensitive to PARP inhibition. ATR inhibitors NU6027 and VE-821 have been shown to increase the cytotoxic activity of PARP inhibitors (rucaparib and veliparib) in two BRCA wild-type cancer cell lines and various ovarian cancer cell lines [35,107]. Whilst the CHK1 inhibitors AZD7762 and LY2603618 have been reported to show cytotoxic synergy with several PARP inhibitors in pancreatic and breast cancer cell lines [108,109,110]. In addition a combination of olaparib and AZD7762 sensitised pancreatic cells to ionising radiation [111]. Finally, a combination of ATR inhibitor (AZD6738) and PARP inhibitor (olaparib) was tolerated in vivo through intermittent doses and showed significant anti-tumour efficacy in multiple human patient derived primary explant models [112].

Targeting enzymes in the same pathway also appears to have a synergistic/synthetically lethal effect. Such findings have been supported by the demonstration of the synergistic cytotoxicity of sub-toxic doses of the ATR and CHK1 inhibitors, VE-821 and AZD7762 in U2OS and MCF-7 cancer cells when combined. Efficacy was also demonstrated in vivo in H460 lung tumour xenografts without affecting animal body weight and no cytotoxicity was seen in non-transformed fibroblast cells highlighting the potential benefit of the combination in the clinical setting [113]. As described above, the kinase Wee1 transduces the signal from CHK1 to inhibit G2/M transition by phosphorylation of CDK1 [23]. The Wee1 inhibitor, MK-1775, has been shown to act synergistically with CHK1 inhibitors AR458323 and PF-00477736 to induce apoptosis in multiple cancer cell lines [114,115]. The combination of MK-1775 and PF-00477736 has also be shown to have an anti-tumour effect in nude mice bearing OVCAR5 xenografts [115].

## 7. Single Agent Activity and Determinants of Sensitivity

### 7.1. Replication Stress

In addition to the potential of ATR and CHK1 inhibitors as chemo- and radiosensitisers, data suggests that they may also have single agent activity through exploitation of certain phenotypic alterations in cancer. Many transforming oncogenes that promote S-phase entry were predicted to cause replicative stress and hence sensitivity to ATR/CHK1 inhibition, which has been borne out experimentally. Myc-driven oncogenic stress in mouse embryonic fibroblast (MEF) cells was shown to lead to synthetic lethality with ATR and CHK1 inhibitors, an effect even more pronounced in p53 deficient cells [116]. Similarly, K-Ras or H-Ras transformation in MEF cells, was synthetically lethal in combination with siRNA knockdown of ATR [117]. The ATR inhibitor, AZD6738, also had single agent activity in a variety of K-Ras mutant NSCLC cell lines [92]. However, in contrast, CHK1 inhibitors caused no detectable impact on γH2AX levels or apoptosis in K-Ras-induced pancreatic adenocarcinomas [116]. Promotion of S-phase entry by induced overexpression of cyclin E1, a feature found frequently in cancer cells (particularly ovarian cancer) was also demonstrated to confer sensitivity to the ATR inhibitor ETP46464, and ETP46464 was also more cytotoxic in tumours derived from Ras and Myc transformed MEF cells [118]. In addition, asynchronous expression of H-Ras, K-ras or c-Myc selectively sensitised cells to ATR inhibition by inhibitor, ATR-45 [119]. In vivo, the ATR inhibitor AZ20 was found to have anti-tumour efficacy against nude mice bearing LoVo colorectal tumour xenografts, with K-Ras mutation and expression of c-Myc, H-Ras, N-Ras, Myb and Fos oncogenes [71]. AZ20 also had anti-tumour activity against AML^MLL^ cells, which contain an activating mutation in N-Ras, growing in immunocompetent mice [120]. In an RNAi screen the tumour suppressor gene ARID1A, recurrently mutated in a variety of tumour types, was found to be synthetically lethal in combination with ATR inhibition. Furthermore, knockdown of ARID1A was able to sensitise human cancer cell lines to VE-821 and VX-970 in vitro and mice with HCT116 xenograft tumours in vivo [121].

Defects in nucleotide biosynthesis and the DNA replication machinery are also responsible for increasing replication stress. Most recently an siRNA library screening approach identified that depletion of *RRM1* and *RRM2*, the ribonucleotide reductase subunits, and *POLD1* and *PRIM1* which encode DNA polymerase δ and DNA primase, as synthetically lethal with ATR [122] and inhibition of DNA polymerase α has also proven to be synergistic with ATR [123]. POLD1 depletion was also shown to significantly increase the sensitivity of DLD1 cells towards the ATR inhibitors NU6027 and VE-821, and the unselective CHK1 inhibitor UCN-01 [122].

### 7.2. DNA Damage Response

The concept of synthetic lethality is already being exploited by PARP inhibitors e.g., olaparib and rucaparib, as inhibition of PARP, an enzyme involved in the base excision repair (BER) pathway, causes increased cytotoxicity in HRR-defective cells. Two genes are described as synthetically lethal if mutation or inactivation of either gene alone has no effect on cellular viability, while simultaneous mutation or inactivation of the genes leads to cell death. In keeping with this, inhibition of ATR and CHK1 (both crucial for HRR) has been shown to be synthetically lethal in cells defective in BER, through inactivation of XRCC1 [35,124,125]. These data, along with numerous other evidence suggest that sensitivity to ATR and CHK1 inhibition can arise from defects in the multiple DNA surveillance and repair pathways.

XRCC1 has also been implicated in nucleotide excision repair (NER) and, ERCC1, a protein involved in resolving bulky adducts, double strand breaks and interstrand crosslinks via NER and other pathways has also been suggested to have a synthetically lethal interaction with not only ATR, but also CHK1 inhibition. Knockdown of both ERCC1 and its binding protein XPF significantly sensitised cells to ATR and CHK1 inhibitor treatment, along with cisplatin treatment. However other proteins of the NER pathway e.g., XPC, XPA and the TFIIH components XPB and XPD were not synthetically lethal with ATR or CHK1 inhibition [126]. Dysfunction of ERCC2, the helicase upstream of ERCC1, caused only a modest sensitisation to ATR inhibition by VE-821 [125]. Defects in both the BER and NER pathways have been reported in a variety of different cancer types highlighting the clinical potential for ATR and CHK1 inhibitors [127,128].

Disruptions in non-homologous end-joining (NHEJ) has also been shown to sensitise cells to ATR/CHK1 pathway inhibition. DNA-PK, the key signalling kinase in NHEJ consists of the DNA binding subunits Ku70 and Ku80 and the catalytic subunit, DNA-PKcs. Knockdown of *XRCC5* (Ku80) and *XRCC6* (Ku70) was identified as synthetically lethal with ATR knockdown [124] and depletion of Ku80 was also shown to confer sensitivity to pharmacological inhibition of ATR (VE-821) and CHK1 (V158411) in CHO cells [125,129]. In marked contrast, loss of DNA-PKcs conferred resistance to ATR and CHK1 inhibition, and upregulation of DNA-PKcs conferred sensitivity [125,129]. Defects in the Fanconi Anaemia (FA) pathway, responsible for DNA repair after treatment with cross-linking agents e.g., cisplatin, have also been shown to increase the sensitivity to CHK1 inhibition. Using isogenic pairs of cell lines differing only in the Fanconi Anemia (FA) DNA repair pathway, it has been shown that FA deficient cell lines were hypersensitive to CHK1 downregulation by siRNA knockdown or pharmacological inhibition by GO6976 and UCN-01. These findings were also confirmed in vivo using whole zebra fish embryos [130] suggesting a role for CHK1 inhibitors in the approximate 15% of all tumours which harbour defects in the FA pathway [131].

As the ATR and ATM mediated pathways function in parallel in the DDR, it has been proposed that defects in ATM pathway signalling genes can confer sensitivity to single-agent ATR inhibition. ATM- or p53-deficient cells were more sensitive to AZD6738 monotherapy compared to ATM/p53-proficient cells in vitro and in vivo [132]. Although there is evidence that ATM defects confer sensitivity to single agent ATR inhibitors and chemosensitisation by ATR inhibitors, such evidence is lacking for CHK1 inhibitors. Somatic *ATM* and *TP53* mutations result in a markedly increased susceptibility to cancers and are commonly found in cancers, therefore these findings allude to an applicability of ATR inhibition to treat multiple cancers.

Finally, defects in the HRR pathway have also been found to increase sensitivity to ATR and CHK1 inhibitors as single agents. CHO cells defective in BRCA2 (involved in RAD51 recruitment to single-stranded DNA) or XRCC3 (which complexes with RAD51) were substantially more sensitive to the ATR inhibitor VE-821 than parental HRR-proficient cells [125]. RAD51 inhibition by BO2 also significantly sensitised cells to the ATR inhibitor VE-821 and CHK1 inhibitor AZD7762 [133]. In addition, in two synthetic lethal screens using the ATR inhibitor VE-821, genes in the ATR/CHK1 pathway itself, including: ATR, ATRIP, RPA, Claspin, Hus1, RAD1 and CHK1 were the strongest hits [126,134].

## 8. ATR and CHK1 Inhibitors in Clinical Trials

The recent FDA approvals of the PARP inhibitors: olaparib (Lynparza™, Astrazeneca, Canbridge UK), rucaparib (Rubraca^®^, Clovis Oncology, Boulder, CO, USA) and niraparib (Zejula™, Tesaro Inc., Waltham, MA, USA) in BRCA mutant high grade ovarian cancer [135,136] prove that the DDR can successfully be exploited to treat cancer. The preclinical data discussed in earlier sections of this review demonstrate that the ATR-CHK1 axis is an attractive target in cancer treatment as chemo- or radio-sensitisers, in combination with molecularly targeted drugs or as single agents in tumours with the appropriate molecular pathology.

This review of the clinical data focussed on recent data with the new, potent and selective inhiitors. There are two ATR inhibitors: VX-970 (recently acquired by Merck KGaA, Darmstadt, Germany, from Vertex pharmaceuticals) and AZD6738 (Astrazeneca) and three novel agents targeting CHK1: SRA737 (previously known as CCT245737, Sierra Oncology Inc., Vancouver, BC, Canada), MK8776 (previously known as SCH-900776, Merck and Co., Whitehouse Station, NJ, USA) and LY2606368 (Prexasertib, Lilly Oncology, Indianapolis, IN, USA) in early phase clinical trial development. Up-to-date review of the clinical trials.gov global database shows 23 registered ongoing studies recruiting patients to both single agent and combination uses (Table 2) (http://www.clinicaltrials.gov). The combination studies include chemotherapy, radiotherapy and other targeted agents. Here we review the limited clinical data available from studies that have completed or reported preliminary findings at international conferences.

VX-970 (VE-822), a potent selective intravenous ATR kinase inhibitor, was the first ATR inhibitor to go into human anti-cancer drug trials [81]. This first phase I trial investigated VX-970 monotherapy and in combination with carboplatin (CP) incorporating pharmacodynamic (PD) studies. In part A, single patient cohorts received VX-970 monotherapy QW; 3 + 3 cohorts were commenced if grade 2 drug-related toxicities were observed. Part B of the study involved 3 + 3 patient cohorts receiving CP on day (D) 1 + VX-970 on D2 and D9 in 21-day cycles. Results from part A (*n* = 17), showed VX-970 to be well tolerated with no dose limiting toxicity (DLT) seen, responses included a patient with heavily pre-treated K-ras wild type metastatic colorectal cancer who had a complete response (RECIST) sustained for >20 months. This patient was subsequently found to have complete loss of ATM (assessed by immunohistochemistry). 5 other patients had stable disease as best response, with median duration of response of 11 weeks. Inhibition of ATR was assessed by measuring changes in levels of phosphorylated-CHK1 (pCHK1) in paired pre-dose/post-dose tumour biopsies in three patients. Results showed >70% reduction in pCHK1 following treatment with VX-970. The recommended phase 2 dose (RP2D) for VX-970 monotherapy was 240 mg/m^2^ once weekly and 240 mg/m^2^ twice weekly. In part B VX-970 was generally well tolerated in combination with CP with mainly grade 1–2 toxicities. CP dose delays and reductions occurred in 6 patients due to neutropenia and/or thrombocytopenia. Clinical data were consistent with toxicity modelling that had predicted probabilities of ≤5% WHO Grade 4 neutropenia and <1% thrombocytopenia at the RP2D which was found to be VX-970 90 mg/m^2^ + Carboplatin AUC5. One patient with germline BRCA1 mutant and platinum-refractory, PARP inhibitor-resistant ovarian cancer with a somatic Y220C TP53 mutation had RECIST partial response for 6 months and 8 other patients in part B had stable disease [137].

Subsequent phase 1 studies have investigated VX-970 in combination with gemcitabine (Gem) (NCT02157792) with the RP2D being VX-970 210 mg/m^2^ and Gem 1000 mg/m^2^ [138] and the combination of VX-970 with cisplatin with the RP2D of VX-970 140 mg/m^2^ and Cisplatin 75 mg/m^2^, with anti-tumour responses seen in platinum-refractory/resistant patients [139] Study NCT02157792 is ongoing with dose expansion cohorts in biomarker-defined non-small cell lung cancer (NSCLC), small cell lung cancer (SCLC) and triple negative breast cancer. Results of the activity of the VX-970 combinations in these patient populations and the VX-970—novel agent combinations listed in Table 2 are eagerly awaited. The future development of VX-970 is uncertain as in late 2016 Vertex pharmaceuticals sold the VX-970 ATR programme to Merck KGaA who now take on all full research and development responsibility.

AZD6738 was the first orally available ATR inhibitor to enter clinical trials and is under investigation as a single agent and in combination in a variety of indications, shown in Table 2. To date there are no published preliminary results and recruitment to each trial is ongoing [140]. Interestingly, these AZ sponsored studies are investigating both the combinations of AZD6738 and the PARP inhibitor olaparib and AZD6738 and the PD-L1 antibody durvalumab (MEDI4736).

Early results from the CHK1 inhibitor trials have shown that this class of drugs are well tolerated and reassuringly the cardiac dose-limiting toxicity that arrested the development of AZD7762 [141] has not been reported in trials involving the more specific CHK1 inhibitors, suggesting an off-target effect [142].These more specific inhibitors include MK-8776, an intravenous, potent, selective CHK1 inhibitor, which has entered phase 1 clinical trials.

A phase 1 trial investigated MK-8776 as monotherapy and in combination with gemcitabine in patients with advanced solid malignancies [143]. Forty-three patients were treated by intravenous infusion with MK-8776 at seven dose levels ranging from 10 to 150 mg/m^2^ as monotherapy and then in combination with gemcitabine 800 mg/m^2^ (part A, *n* = 26) or gemcitabine 1000 mg/m^2^ (part B, *n* = 17). Forty percent of patients had three or more prior treatment regimens, and one third of patients had previously received gemcitabine. As monotherapy, MK-8776 was well tolerated, with the most common adverse events being QTc prolongation (19%), nausea (16%), fatigue (14%), and constipation (14%). Combination therapy demonstrated a higher frequency of adverse effects, predominantly fatigue (63%), nausea (44%), decreased appetite (37%), thrombocytopenia (32%), and neutropenia (24%), as well as dose-related, transient QTc prolongation (17%). The median number of doses of MK-8776 administered was five doses, with relative dose-intensity of 0.96. Bioactivity was assessed by γ-H2AX ex vivo assay. Of 30 patients evaluable for response, two showed partial response, and 13 exhibited stable disease. The RP2D is MK-8776 200 mg plus gemcitabine 1000 mg/m^2^ on days 1 and 8 of a 21-day cycle.

MK-8776, then known as SCH 900776, has also been investigated in a phase 1 trial with cytarabine [144]. In this phase 1 trial twenty-four adults with relapsed/refractory acute leukaemias received timed sequential, continuous infusion cytarabine 2 g/m^2^ over 72 h (667 mg/m^2^/24 h) beginning on day 1 and again on day 10. SCH 900776 was administered as a 15- to 30-min infusion on days 2, 3, 11, and 12. The starting dose of SCH 900776 was 10 mg/m^2^/dose. DLT were QT interval prolongation and grade 3 palmar-plantar erythrodysesthesia which occurred at 140 mg flat dosing (dose level 5, equivalent to 80 mg/m^2^. Complete remissions were observed in 8 of 24 (33%) patients. On study marrow blasts obtained pre-treatment and during SCH 900776 dosing showed increased phosphorylation of H2AX signalling unrepaired DNA damage. The Phase 2 study of the combination versus single agent cytarabine are now awaited.

The only other CHK1 inhibitor with reported clinical trial results to date is LY2603618. LY2603618 is a selective inhibitor of CHK1 which has been investigated in two phase 1 trials in combination with gemcitabine [145,146] and subsequently entered phase 2 trials. This phase 2 trial assessed the overall response rate, safety, and pharmacokinetics (PK) of LY2603618 and pemetrexed in patients with NSCLC progressing after a prior first-line treatment regimen (not containing pemetrexed) [147]. Expression of p53, as measured by immunohistochemistry and genomic variant analysis, was assessed as a predictive biomarker of response. Fifty-five patients were enrolled in the study. No patients experienced a complete response; a partial response was observed in 5 patients (9.1%; 90% CI, 3.7–18.2) and stable disease in 20 patients (36.4%). The median progression-free survival was 2.3 months (range, 0–27.1). Safety and PK of LY2603618 in combination with pemetrexed were deemed favourable. Interestingly no association between p53 status and response was observed and the response rates were comparable with historical single agent pemetrexed data. So this combination has not been further investigated.

There are currently 23 registered clinical trials investigating ATR and CHK1 inhibitors. The field is expanding rapidly and the real challenges moving forward will be how to identify the select group of patients who will benefit from ATR/CHK1 inhibitor monotherapy and working out the correct schedule and dosing in combination with both traditional chemotherapies and other novel agents to avoid toxicity but enhance responses. We eagerly await the results of the many ongoing studies.

## 9. Summary

The development of increasingly potent and specific inhibitors of ATR and CHK1 has allowed their role to be explored extensively in the pre-clinical setting. ATR and CHK1 are clearly good targets for the selective sensitisation of cancers to conventional therapy. Interestingly, although inhibitors of both kinases sensitise cells to antimetabolites, exemplified largely by gemcitabine, the sensitisation of cisplatin is more evident with ATR inhibitors and sensitisation of taxanes has only been reported with CHK1 inhibitors. Both ATR and CHK1 inhibitors as single agents exploit the specific molecular pathologies of cancer, in particular the high levels of replication stress resulting from activated oncogenes. The role of p53 status in not altogether clear: where matched cell lines have been used in general those lacking p53 function have shown the greatest sensitivity to ATR and CHK1 inhibitors however single agent activity and chemosensitisation is evident in cells with wt p53, most probably because of the multiple derangements that can compromise G1 cell cycle control. As potent and specific ATR and CHK1 inhibitors enter clinical evaluation, with promising early results, we are learning more about this exciting class of therapeutic agents.

## Figures and Tables

**Table 1:** Potent and specific small molecule inhibitors of ATR and CHK1.

**CHK1 Inhibitors**			
**Name**	**Structure**	**IC_50_/Ki**	**Specificity**
AZD7762	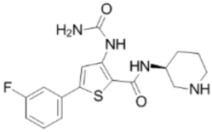	CHK1 IC_50_ = 5 nM	Equally potent: CHK1/CHK2
V158411	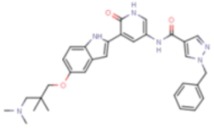	CHK1 IC_50_ = 4.4 nM	Equally potent: CHK1/CHK2; 20-fold CHK1 vs. CHK2 in cells (Cellular CHK1 IC_50_ = 48 nM vs. CHK2 IC_50_ = 904 nM)
PF477736	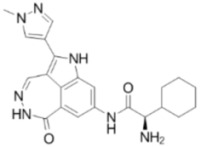	CHK1 Ki = 4.9 nM	100-fold CHK1 vs. CHK2
MK8776/SCH900776	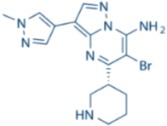	CHK1 IC_50_ = 3 nM	500-fold CHK1 vs. CHK2
CCT244747	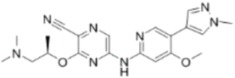	CHK1 IC_50_ = 8 nM	>1000-fold CHK1 vs. CHK2
CCT245737	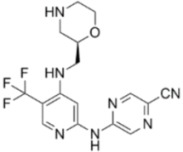	CHK1 IC_50_ = 1.3 nM	>1500-fold CHK1 vs. CHK2
LY2603618	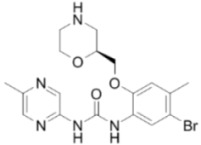	CHK1 IC_50_ = 7 nM	>1500-fold CHK1 vs. CHK2
**ATR Inhibitors**			
**Name**	**Structure**	**IC_50_/Ki**	**Specificity**
NU6027	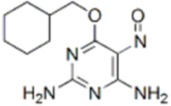	ATR IC_50_ = 1 nM	ATR, CDK1 (Ki = 2.5 µM), CDK2 (Ki = 1.3 µM)
ETP-46464	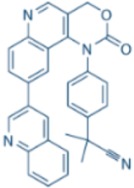	ATR IC_50_ = 25 nM	ATR
VE-821	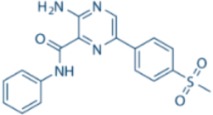	ATR IC_50_ = 26 nM	>100-fold ATR vs. ATM/DNA-PK
VE-822/VX-970	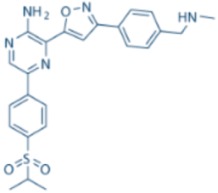	ATR IC_50_ = 0.2 nM	>100-fold ATR vs. ATM/DNA-PK
AZ20	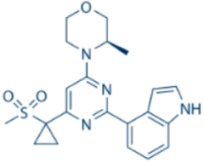	ATR IC_50_ = 5 nM	>600-fold ATR vs. ATM/DNA-PK/PI-3K
AZD6738	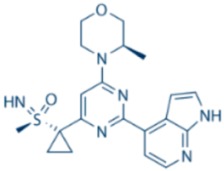	ATR IC_50_ = 1 nM	ATR

**Table 2:** Open and actively recruiting trials using ATR and CHK1 inhibitors (February 2017).

Target, Agent	Phase	Combination	Indication	NCT No.
ATR-VX-970, intravenous, Vertex pharmaceuticals (recently licenced to Merck KGaA, Germany)	1	irinotecan	Advanced solid tumours	NCT02595931
	1	Veliparib + Cisplatin	Advanced solid tumours	NCT02723864
	1/2	Topotecan	Advanced small cell lung, cervical, endometrial, ovarian cancers	NCT02487095
	2	Gemcitabine	Advanced ovarian/fallopian tube/primary peritoneal cancer(OC/FT/PP)	NCT02595892
	2	Carboplatin/Gemcitabine	Advanced OC/FT/PP	NCT02627443
	2	Cisplatin/Gemcitabine	Advanced urothelial cancers	NCT02567409
	1	Cisplatin/Radiotherapy	Locally dvanced HPV negative SCC head and neck cancers	NCT02567422
	1	Whole brain radiotherapy	Non- small cell lung cancers with brain mets	NCT02589522
	1	Gemcitabine, Cisplatin, Etoposide, Carboplatin	Multiple parts including p53mut NSCLC, triple negative breast cancers	NCT02157792
ATR—AZD6738, oral, Astrazeneca	1	Single agent and in combination with radiotherapy	Advanced solid tumours	NCT62223923
	1	Paclitaxel	Advanced solid tumours	NCT02630199
	1	Carboplatin, Olaparib, MEDI4736	Advanced solid tumours	NCT02264678
CHK1—LY2606368, (Prexasertib), intravenous, Eli Lilly	1	Cytarabine and Fludarabine	Relapsed/Refractory Acute Myelogenous Leukemia (AML) and High-Risk Myelodysplastic Syndrome	NCT02649764
	1	^14^C radiolabelled LY2606368	Advanced solid tumours	NCT02778126
	1	Single agent	Japanese patients with Advanced solid tumours	NCT02514603
	2	Single agent	Advanced small cell lung cancer	NCT02735980
	1	Ralimetinib (p38 MAPK inhibitor)	Advanced solid tumours	NCT02860780
	1	Cisplatin, Cetuximab, Intensity Modulated Radiation Therapy	Advanced solid tumours, Head and Neck	NCT02555644
	1	Cisplatin, Cetuximab, Pemetrexed, Fluorouraci	Advanced solid tumours	NCT02124148
	2	Single agent	BRCA1/2 Mutation Associated Breast or Ovarian Cancer, Triple Negative Breast Cancer, High Grade Serous OC, and Metastatic CRPC	NCT02203513
	2	Single agent	Advanced Solid Tumours Exhibiting Replicative Stress or Homologous Recombination Repair Deficiency	NCT02873975
CHK1—SRA 737 previously known CCT245737, Sierra Oncology Inc.	1	Gemcitabine + Cisplatin or Gemcitabine Alone	Advanced solid tumours	NCT02797977
	1	Single agent	Advanced solid tumours	NCT02797964
